# International and National Expert Group Evaluations: Biological/Health Effects of Radiofrequency Fields

**DOI:** 10.3390/ijerph110909376

**Published:** 2014-09-10

**Authors:** Vijayalaxmi, Maria R. Scarfi

**Affiliations:** 1 Department of Radiology, University of Texas Health Science Center, San Antonio, TX 78299, USA; 2 CNR-Institute for Electromagnetic Sensing of Environment, Napoli 80124, Italy; E-Mail: scarfi.mr@irea.cnr.it

**Keywords:** radiofrequency fields, biological effects, expert committees, evaluations

## Abstract

The escalated use of various wireless communication devices, which emit non-ionizing radiofrequency (RF) fields, have raised concerns among the general public regarding the potential adverse effects on human health. During the last six decades, researchers have used different parameters to investigate the effects of* in vitro* and* in vivo* exposures of animals and humans or their cells to RF fields. Data reported in peer-reviewed scientific publications were contradictory: some indicated effects while others did not. International organizations have considered all of these data as well as the observations reported in human epidemiological investigations to set-up the guidelines or standards (based on the quality of published studies and the “weight of scientific evidence” approach) for RF exposures in occupationally exposed individuals and the general public. Scientists with relevant expertise in various countries have also considered the published data to provide the required scientific information for policy-makers to develop and disseminate authoritative health information to the general public regarding RF exposures. This paper is a compilation of the conclusions, on the biological effects of RF exposures, from various national and international expert groups, based on their analyses. In general, the expert groups suggested a reduction in exposure levels, precautionary approach, and further research.

## 1. Introduction

The introduction of mobile phones emitting non-ionizing radiofrequency (RF) fields and delivering voice, data and images has increased concern in the general public regarding the potential adverse health effects from RF exposure, especially the development of brain cancer since the antenna is held close to head when the phone is being used. During the last several decades, numerous researchers have been examining the biological and health effects of acute and long-term* in vitro* and* in vivo* RF exposure in animals and humans or cells. These included: (i) epidemiological studies in humans examining the incidence of brain and other types of cancers, (ii) carcinogenesis in normal, transgenic and tumor-prone animals, (iii) genetic damage (excess DNA damage in somatic cells, if un-repaired and/or mis-repaired, can lead to carcinogenesis while similar damage in germ cells can be transmitted to the next generation), and (vi) non-genotoxic indices such as cell cycle/proliferation, apoptosis, inter-/intra-cellular signaling, gene and protein expression, and oxidative stress as well as immune, reproduction (development, teratology), neurological (blood-brain barrier, behavior, hypersensitivity) responses. The data reported from each of these investigations published in peer-reviewed scientific journals indicated both an absence and a presence of an effect from RF exposure [1,2,3,4,5,6,7,8,9,10,11].

For human health risk assessment, it is essential to use the “weight of scientific evidence” based on the quality of published studies which should include detailed description of RF dosimetry, exposure conditions and protocols consistent with good laboratory practices, sample sizes with sufficient statistical power, as well as confirmation and replication studies conducted by independent researchers. International organizations, such as the Institute of Electrical and Electronic Engineers (IEEE) and the International Commission on Non-Ionizing Radiation Protection (ICNIRP) have considered all of the available peer-reviewed scientific literature and used the weight of scientific evidence approach to set-up the guidelines or standards for RF exposures in occupationally exposed individuals and the general public to protect against established adverse effects [12,13,14].

Thus far, the most robust effects of RF exposure were observed when the whole body averaged specific absorption rate (SAR) exceeded 4 W/kg which was associated with heating and raised the body temperature by about 1 °C in animals (rats and monkeys): this information was used to set up guidelines or standards to protect people from undue RF exposure. A safety factor of 10x lower SAR (0.4 W/kg) was included to allow for thermal, environmental and possible long-term effects in occupational exposures. A further safety factor of 5× lower SAR (0.08 W/kg, total 50× lower) was introduced to provide adequate margin to protect the general public and persons with potentially different sensitivities, such as infants and elderly. For localized exposures, protection of eye injury has been used to set a limit of 10 W/kg for the workers and 2 W/kg for the general public, both averaged over 10 gram tissue (10× and 50× below the threshold level, above that could cause cataracts in rabbits). Since guidelines and standards (up-dated as and when new peer-reviewed scientific data were available) were based on rigorous, comprehensive reviews and weight of scientific evidence, a great majority of the countries in the world have adopted them to protect occupationally exposed individuals as well as the general public from RF exposures. Scientists who are “experts” in various countries have also considered these guidelines and standards to provide the required scientific information for policy-makers to develop and disseminate authoritative health information to the general public regarding RF exposures. The aim of this review is to compile the conclusions of various international and national expert groups based on their analyses and, are listed in alphabetical order below (the reports available in English language only were considered for this review). Much of the text in *italics* below was the information, as presented, in the various evaluations/reports.

## 2. Evaluations

The members serving in the “expert groups” (EGs) were selected by the health agencies and relevant authorities in different countries based on their expertise in RF dosimetry, biology, epidemiology, medicine, and social issues and peer-reviewed scientific publications. Basically, the EGs have carefully examined and evaluated all of the data published, in peer-reviewed scientific journals, for various parameters/endpoints in animals and humans exposed* in vitro* and* in vivo* to RF fields. Generally, a well-defined criteria/protocol was used in the evaluation process: whether or not the publications/investigations have included detailed RF dosimetry, appropriate experimental groups in the study/laboratory protocols, adequate sample size, consistency in the results and statistical analyses, presence of confounders and potential sources of bias, confirmation and replication studies,* etc.* Some EGs, for example: (i) the European health risk assessment network on electromagnetic fields exposure (EFHRAN, section 2.1.5) have used the evaluations for each end-point based on sufficient, limited, inadequate or inconsistent/lack of evidence and (ii) the Scientific Committee on Emerging and Newly Identified Health Risks (SCENIHR, section 2.1.4) also included whether or not a particular publication should not be considered in the review process and the reason(s) for doing: consequently, not all papers were given the same weight in the risk assessment. All of these criteria were included in the overall “weight of scientific evidence” for adverse effects, if any, due to RF exposure. The detailed evaluations are available in public domain (pdf format and/or as information sheets/statements/released to the press) in the country’s website to keep the public informed about the RF exposure guidelines, as well as latest developments in RF research. Some of the information might have been changed and/or updated.

### 2.1. International Organizations

A summary of different international EGs evaluations together with the topics discussed and the final recommendations/advises are presented in Table 1.

#### 2.1.1. International Agency for Research on Cancer (IARC) 

The International Agency for Research on Cancer (IARC, Lyon, France) [15] is the specialized cancer agency of the World Health Organization. In 2008, IARC has published the World Cancer Report [16] and stated: “*Radiofrequency radiation emitted by mobile telephones has been investigated in a number of studies. There is some evidence that long-term and heavy use of mobile/cellular phones may be associated with moderate increased risks of gliomas, parotid gland tumours, and acoustic neuromas; however, evidence is conflicting and a role of bias in these studies cannot be ruled out. With reference to radio frequency, available data do not show any excess risk of brain cancer and other neoplasms associated with the use of mobile phone*”. Regarding brain tumors, IARC report [16] also stated: “*After 1983 and more recently during the period of increasing prevalence of mobile phone users, the incidence has remained relatively stable for both men and women*”. 

**Table 1:** Summary of international expert group evaluations on the biological and health effects reported in all animal and human cells (including human epidemiological investigations) exposed *in vitro* and* in vivo* to non-ionizing radiofrequency fields.

International Organization	Expert Group. Literature Evaluated. Year.	Conclusions	Recommendation	Citations (see the text for details)
IARC	All topics. 2011.	No increased risk for meningioma and glioma with mobile phone use. Increased risk of glioma at the highest cumulative hours of mobile phone use. Limited evidence from animal studies. Weak evidence from other relevant studies.	RF is a possible carcinogen, class 2-B.	[29]
IEEE	COMAR. All topics. 2009.	Public health officials should continue to use RF safety limits of international organizations.	See the text for exposure guidelines.	[40]
ICNIRP	All topics. 2009.	Impossible to disprove non-thermal effects. Poor evidence for chronic/low-level effects. Studies with adequate RF exposure assessment did not reveal any health-related effects.	See the text for exposure guidelines.	[42]
EU	SCENIHR. All topics. 2013.	No consistent evidence on cognitive function. No clear effect on neurological diseases. Unequivocal evidence on head/neck and childhood cancers. *In vivo* studies in animals were negative. No* in vitro* effects below the exposure guidelines. Uncertainties remain.	A total of 37 recommendations made for future research with high, medium and low priorities.	[45,46,47]
EU	EFHRAN. All topics. 2012.	No evidence for electromagnetic hypersensitivity. Limited evidence for stress response genes* in vitro*. Inadequate evidence for cancer and neurological diseases.	-	[49]
WHO	All topics. 2011.	IARC recommendation of RF as class 2-B carcinogen, a category used when a causal association is considered credible but, chance, bias or confounding factors cannot be ruled out with reasonable confidence.	Studies on long-term mobile phone use, especially among young people.	[51,52]
	Base Stations and Accidents. 2013.	Increased traffic accidents due to mobile phone use during driving.	Further research is warranted	

**Figure 1:**
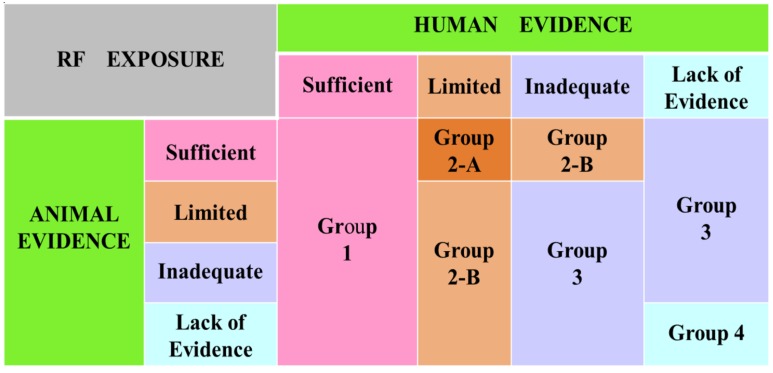
International Agency for Research on Cancer (IARC) classification of environmental agents in different categories, based on the “weight of scientific evidence”.

Note: Other relevant and mechanistic data are important when the data in humans are inconclusive.

The core portfolio of IARC’s activities is the program on monographs. For this, the agency seeks scientists with significant peer-reviewed scientific publications/expertise who will serve as members in working groups (WG), search all peer-reviewed scientific literature, prepare a critical review, discuss and combine all relevant information to evaluate the weight of evidence of the agent in question cause carcinogenesis in humans. The final consensus evaluations/analyses were placed in one of the following five categories (groups 1, 2-A and 2-B, 3 or 4; Figure 1). Group 1: Carcinogenic (sufficient evidence in human epidemiological/clinical studies irrespective of other evidences). Group 2A: Probably carcinogenic (limited evidence from human epidemiological/clinical studies, and sufficient evidence from the animal investigations. Group 2B: Possibly carcinogenic (limited evidence from human epidemiological/clinical studies and inadequate evidence in animal models). Group 3: Not classifiable (inadequate evidence from human epidemiological/clinical studies as well as inadequate evidence from animal studies). Group 4: Probably not carcinogenic (lack of evidence from both human epidemiological/clinical and animal studies). Thus far, IARC has classified a total of ~970 agents (113, 66, 285, 505, and 1, in the groups 1, 2A, 2B, 3, and 4, respectively, monograph volumes 1-109). The complete list can be down-loaded from the Internet [17].

In May 2011, an interdisciplinary expert WG of 30 scientists from 14 countries met at IARC to evaluate the carcinogenic risk from RF emitted from mobile communication devices which was based on the rigorous, arduous and “weight of evidence” approach. The four working groups considered the data in all peer-reviewed publications: (i) dosimetry and exposure, (ii) epidemiological studies in humans including the data from The Interphone study group [18], (iii) acute and long-term cancers in experimental animals, and (iv) mechanistic and other relevant information. The conclusions were as follows. (i) Epidemiological studies indicated no increased risk for meningioma and glioma with mobile phone use while there was an increased risk of glioma at the highest cumulative hours of mobile phone use. (ii) Studies in experimental animals and the mechanistic/other relevant end-points/parameters showed that the evidence for RF-induced carcinogenesis was “limited” and “weak”, respectively. Considering all of the evidence together, RF was classified as possible carcinogen in group 2B and released the information to the press [19]. A summary report was published after the meeting [20]. Subsequent meta-analysis of the data in human cells only (reported in 88 peer-reviewed scientific publications during 1990–2011) did not suggest a genotoxicity-based mechanistic evidence to classify RF as 2B carcinogen [21]. Furthermore, the overall brain cancer indices among the general population did not suggest an increasing trend after the introduction of mobile phones [22,23,24,25]. A more recent prospective study also revealed significantly decreased risk for glioma in mobile phone users [26]. A potential hypothesis of RF-induced “adaptive response” has been proposed recently for the observed decreased incidence of brain cancer in mobile phone users [27,28]. The detailed IARC evaluations were published in monograph #102 [29]. 

Several national and international organizations commented on IARC evaluation of RF exposure as class 2-B carcinogen to humans. Australia: Radiation Protection and Nuclear Safety Agency (ARPANSA) [30]: *“…welcomes the report and considers that the classification by IARC corresponds to the current ARPANSA advice, including its advice on practical ways in which people can reduce their exposure to the electromagnetic fields produced by wireless telephones”*. ARPANSA has also recommended “*parents should encourage their children use the methods to reduce exposure*”. The Australian Cancer Council [31]: *“These findings show limited evidence linking mobile phones to glioma and acoustic neuroma and inadequate evidence to draw conclusions for any other types of cancer. However, it does sound a warning bell and highlights the need more research in this area”*. Health Canada [32]: *“IARC’s recent classification of RF energy as “possibly carcinogenic to humans” is an acknowledgement that limited data exists that suggests RF energy might cause cancer”*. *“At present, the scientific evidence is far from conclusive and more research is required”*. The consumers can “*Limit the length of cell phone calls; Replace cell phone calls with text messages or use “hands-free” device; Encourage children under the age of 18 to limit their cell phone usage”*. ICNIRP [33]: *“…awaits with interest the full Monograph that explains the justification and arguments put forward by IARC in arriving at this conclusion”*. Spain (CCARS) [34]: *“it does not either establish or quantify the degree of risk whilst recommending further research to confirm this hypothesis”*.* “The CCARS will analyze the contents of the Monograph in great detail once it is published. On the basis of this risk analysis, it will then make available to the relevant public health authorities the knowledge and evidence required to assess the need to adopt informational and preventive measures regarding the use of RF”*. UK, Health Protection Agency [35]: *“HPA advice is that there is no clear scientific evidence of a cancer risk from exposure to radiofrequencies at levels below international guidelines but the possibility remains. The HPA has always advocated some precaution in the use of mobile phones in case there are long term effects which are presently unknown. Exposures from Wi-Fi equipment are much less than from mobile phones, and are well within international guidelines, so there is no reason why schools and others should not continue to use the technology”*. American Cancer Society, USA [36]: *“At first glance, these new recommendations are very much in line with the American Cancer Society’s current information that the evidence is limited, that further research is needed, and that there are things people who are concerned about radiofrequency exposure can do to limit their exposure, including using an ear piece and limiting cell phone use, particularly among children”*.* “It’s also important to put this 2B classification into perspective. Many common exposures are classified in Category 2B, including gasoline exhaust and even coffee”*. National Cancer Institute, USA [37]: *“…is neither new research nor at odds with previous findings. Both IARC and NCI recommend continued monitoring of both brain cancer trends and new evidence from studies in humans and laboratory animals. In particular, it will be important to assess risk after long-term use, and for younger users*. World Health Organization (WHO) [38]: *“…there is some evidence linking mobile phones to cancer, but it is too weak to make any strong conclusions. Specifically, IARC’s panel said that the evidence that mobile phones pose a health risk was “limited” for two types of brain tumours—glioma and acoustic neuroma—and “inadequate” when it comes to other types of cancer”*.

#### 2.1.2. Institute of Electrical and Electronic Engineers (IEEE) 

The Institute of Electrical and Electronic Engineers (IEEE) [39] is the world’s largest professional association dedicated to advancing technological innovations for the benefit of humanity. It develops standards for a wide range of industries including power and energy, telecommunications and nanotechnology. Its International Committee on Electromagnetic Safety (ICES) has five subcommittees, of which members have wide range of expertise in electrical/electronic/mechanical engineering, computer science, biology, psychology, medicine, and physics: they perform research, evaluation of peer-reviewed scientific publications and develop open consensus and rational safety standards with respect to human exposures in frequency range from 0–300 GHz. The committee that developed the latest version of the RF safety standard IEEE C95.1-2005 had a wide range of participation by experts in engineering, biology, medicine, measurements, and safety programs. In terms of stakeholders, the committee consists of members of the government, military, academia, industry, and general public. The exposure limits were developed by an international committee of more than 125 members representing 25 countries. The technical committee, Committee on Man and Radiation (COMAR), considers that the scientific literature related to biological effects of RF is highly diverse, both in terms of scientific quality and in terms of relevance to possible health and safety risks to humans. Consequently, in its review process, only the studies that met selection criteria which included adequate dosimetry and experimental design, and independent confirmation of reported effects were considered. COMAR [40] concurs with the conclusions of several reviews including those from standard-setting organizations and responsible health agencies in various national governments and, recommended: *“…public health officials continue to base their policies on RF safety limits recommended by established and sanctioned international organizations such as the Institute of Electrical and Electronics Engineers International Committee on Electromagnetic Safety and the International Commission on Non-Ionizing Radiation Protection, which is formally related to the World Health Organization”*.

#### 2.1.3. International Commission on Non-Ionizing Radiation Protection (ICNIRP)

The International Committee on Non-Ionizing Radiation Protection (ICNIRP, Oberschleissheim, Germany) [41] consists of a main commission and various subcommittees examining epidemiology, biology, physics and optics. It works in close collaboration with the World Health Organization (WHO) and publishes guidelines, statements and documents, which are exclusively based on peer-reviewed scientific literature. Hence, a great majority of countries in the world adopt ICNIRP recommended guidelines for exposures to non-ionizing electromagnetic fields. Regarding the frequencies of electromagnetic fields up to 300 GHz, ICNIRP stated [42]: *“...it is the opinion of ICNIRP that the scientific literature published since the 1998 guidelines has provided no evidence of any adverse effects below the basic restrictions and does not necessitate an immediate revision of its guidance on limiting exposure to high frequency electromagnetic fields”*.* “With regard to non-thermal interactions, it is in principle impossible to disprove their possible existence but the plausibility of the various non-thermal mechanisms that have been proposed is very low. In addition, the recent in vitro and animal genotoxicity and carcinogenicity studies are rather consistent overall and indicate that such effects are unlikely at low levels of exposure”*. Furthermore*, “Epidemiological data on possible health effects of chronic, low-level, whole-body exposure in the far-field of radiofrequency (RF) transmitters are poor, especially because of lack of satisfactory individual exposure assessment. The few studies with adequate exposure assessment did not reveal any health-related effects. Exposure levels due to cell phone base stations are generally around one-ten-thousandth of the guideline levels”*. In a review on “Exposure to high frequency electromagnetic fields, biological effects and health consequences (100–300 GHz)” ICNIRP also stated [14]: *“The mechanisms by which RF exposure heats biological tissue are well understood and the most marked and consistent effect of RF exposure is that of heating, resulting in a number of heat-related physiological and pathological responses in human subjects and laboratory animals. Heating also remains a potential confounder in in vitro studies and may account for some of the positive effects reported”.* Regarding mobile phones, brain tumors and the interphones study, the ICNIRP standing committee on epidemiology stated [43]: *“Although there remains some uncertainty, the trend in the accumulating evidence is increasingly against the hypothesis that mobile phone use can cause brain tumors in adults”*.

#### 2.1.4. European Commission (SCENIHR, Scientific Committee for the Emerging and Newly Identified Health Risks)

The European Commission [44] relies on three independent scientific committees to deal with questions related to broad, complex or multidisciplinary issues requiring comprehensive risk assessment of exposures to several new and emerging technologies. The expert members SCENIHR reviewed all peer-reviewed publications covering the entire electromagnetic spectrum according to well-defined criteria. Explanations were also provided whether or not to considering a particular publication in the review process: hence, not all papers were given the same weight in the risk assessment. In 2009, the opinion of the SCENIHR scientific committee on RF stated [45] *“It is concluded from three independent lines of evidence (epidemiological, animal and in vitro studies) that exposure to RF fields is unlikely to lead to an increase in cancer in humans”*.* “...the conclusion that scientific studies have failed to provide support for an effect of RF fields on self-reported symptoms still holds”*.* “There is some evidence that RF fields can influence EEG patterns and sleep in humans. However, the health relevance is uncertain and mechanistic evidence is lacking”*.* “Other studies on functions/aspects of the nervous system, such as cognitive functions, sensory functions, structural stability, and cellular responses show no or no consistent effects”*.* “Recent studies have not shown effects from RF fields on human or animal reproduction and development. No new data have appeared that indicate any other effects on human health”*.

In 2013, the opinion of the expert group of the most recently updated preliminary draft report on RF stated [46,47]:* “Studies on cognitive functions in humans lack consistency; Studies on neurological diseases and symptoms show no clear effect; Human studies on child development and behavioral problems provide only weak evidence; Epidemiological studies do not unequivocally indicate increased risk of brain and other cancers in head and neck region or other malignant diseases including childhood cancer; In vivo studies using a wide variety of animal models have been mostly negative in outcome; Large number of in vitro studies on genotoxic as well as non-genotoxic end-point showed no effects at below the exposure limits: in some cases DNA strand breaks and spindle disturbances were observed”*. A number of areas were identified for future research where the information regarding health effects is either absent or insufficient, or is too discordant to allow science-based assessment of the possibility of health effects. A total of 37 recommendations were made for future research and were listed as high, medium, and low priorities.

#### 2.1.5. European Health Risk Assessment Network (EFHRAN)

Scientists belonging to research institutes from seven European countries (Denmark, France, Hungary, Italy, Slovenia, Spain, and the UK), external collaborators from 12 countries and some industrial groups were associated with EFHRAN project [48], which was funded by the European Commission (EC) to provide advice on policy development for the European Union. Several reports have already been published and all can be downloaded from the Internet [49]. The overall conclusions were that there were no well-established positive effects of low-level RF exposure (SAR < 2 W/kg) in* in vitro* and* in vivo* animals studies. In human risk analysis, the evidence was inadequate for cancer, neurodegenerative and cardiovascular diseases, reproductive outcomes and, lack of effect on hypersensitivity. In addition, the public’s perception of possible health risks due to EMF exposure levels within international guidelines did not necessarily reflect the scientific community’s assessment and, there was a lack of evidence that could support this suggestion. The policy and health authorities in Europe need to invest in improving communication strategies related to EMF, allowing Europeans to have access to high quality and referenced information about the scientific state of the art on EMF and health issues.

#### 2.1.6. The World Health Organization (WHO) 

WHO [50] had established the EMF project in 1996 with several objectives including: (i) develop a solid scientific literature base regarding the potential risks of exposure to EMF, identify gaps in knowledge requiring further research; (ii) facilitate dialog between stakeholders by providing clear and unbiased information on the current scientific knowledge; (iii) help countries to set their national EMF legislation and regulations, and to promote high level of heath protection to all people. In 2011, in fact sheet#193, WHO stated [51]: *“To date, research does not suggest any consistent evidence of adverse health effects from exposure to radiofrequency fields at levels below those that cause tissue heating. Further, research has not been able to provide support for a causal relationship between exposure to electromagnetic fields and self-reported symptoms, or “electromagnetic hypersensitivity”.* Referring to the IARC classification of RF as class 2B carcinogen, in the same fact sheet #193, WHO also stated [51]:* “While an increased risk of brain tumors is not established, the increasing use of mobile phones and the lack of data for mobile phone use over time periods longer than 15 years warrant further research of mobile phone use and brain cancer risk”*. More recently, in 2013, in answers to questions, WHO indicated [52]: *“Studies to date provide no indication that environmental exposure to RF fields, such as from base stations, increases the risk of cancer or any other disease”*.* “Scientists have reported other health effects of using mobile phones including changes in brain activity, reaction times, and sleep patterns. These effects are minor and have no apparent health significance”*.* “Research has shown an increased risk of traffic accidents, some 3–4 times greater chance of an accident, when mobile phones (either handheld or with a hands-free kit) are used while driving due to distraction”*.

### 2.2. National Organizations

A summary of different national EGs evaluations together with the topics discussed and the final recommendations/advises are presented in Table 2.

**Table 2:** Summary of international expert group evaluations on the biological and health effects reported in all animal and human cells (including human epidemiological investigations) exposed *in vitro* and* in vivo* to non-ionizing radiofrequency fields.

Country	Expert Group. Literature Evaluated. Year.	Conclusions	Recommendation	Citations (see the text for details)
Australia	ARPANSA. All topics. 2012–2014	No substantiated evidence for health risk for people living near base stations. Insufficient evidence for higher risk for children. No need to reconsider exposure limits.	Precautionary approach. Reduce exposures. Use hands-free devices.	[55,56,57,58,59]
Belgium	Superior Health Council. All topics. 2011–2014	No proven health risks. Long-term health risks cannot be ruled out.	Limit call numbers/time. As of March 2014, mobile phones designed for young children may not be sold in the market.	[61,62,63]
Canada	Health Canada. All topics. 2012–1014	Cell phone towers are not dangerous. No evidence of adverse effects from WiFi. Since the last revision of safety code 6, no new adverse health effects have been established.	Practical measures to reduce exposures.	[65,66,67]
Finland	STUK. Some topics. 2008–2014	Mobile phone use is not detrimental to health.	Precautionary measures. Not to totally prohibit children to use mobile phones.	[70,71,72,73]
France	ANSES. All topics. 2013	No new proven health effects.	Limit call numbers/time for children and heavy users.	[75,76,77]
Germany	SSK. All topics. 2011	Discrepancy between scientific evidence and risk perception. No overall risks.	-	[79]
	DMF/BFS. All topics. 2011	Risk perception is linked to media coverage.	-	[82]
	Julich Res Institute. Children. 2009	No indications of adverse health effects in children.	-	[84]
Latin America	All topics. 2010	Thermal and non-thermal mechanisms were considered. Insufficient evidence for adverse health effects from* in vitro* and* in vivo* studies.	Precaution principle. Advantages of mobile Phones highlighted.	[85]
Netherlands	Health Council. All topics. 2009–2013	No evidence for medically unexplained symptoms. Limited data do not indicate adverse effect on brain and health of children. Insufficient and inconsistent association of tumors in brain and other regions of head.	No reason to recommend different exposure limits for children than for adults.	[87,88,89]
New Zealand	NRL. All topics. 2008	No health problems when complied with international guidelines. A matter of informed choice for children’s use.	Methods to reduce exposures.	[91,93]
Nordic Countries	Denmark, Finland, Iceland, Norway and Sweden. All topics. 2009–2013	No scientific evidence for adverse health effects. If the number of fixed antennas is reduced, mobile phone will need to use higher power to maintain the connection, thereby the exposure of the general public may increase (2009). To date, do not show adverse health effects below the guidelines or limits adopted in the Nordic countries. (2013).	No need to further limit exposure from WLAN and base stations.	[95]
Norway	NIPH. All topics. 2012	No evidence that weak RF fields cause adverse health effects. Uncertainty in risk assessment is small.	Precaution approach.	[97]
Spain	CCARS. WiFi. 2011	To date, no scientific evidence that exposure to the low emissions levels of these systems produces adverse health effects in school children.	No reason that WiFi systems should not be used.	[99,100]
Sweden	SSM/SSI. All topics. 2008–2014	Potential heating is the source for artifacts. The observed cancer risk estimates below the unity may indicate a “protective effect”. Some repetition studies were conducted. No adverse effects were reported. Most do not support earlier adverse effects.	More research is needed in children.	[102,103,105,106,107,109]
	FAS. All topics. 2012	Overall data do not support increased cancer risk in mobile phone users. No new interaction mechanisms.	Methods to reduce exposure levels.	
Switzerland	FOEN. All topics. 2012	No new confirmed health effects. “Absence of proof of health risks” does not automatically mean proof of their absence.	Precautionary measures. Further research.	[111]
Tanzania	TCRA. All topics. 2010	No substantial evidence for harmful health effects. Many benefits of modern technology.	-	[113]
UK	HPA/PHE. All topics. 2012–2013	No convincing evidence in adults or children for adverse effects below the recommended/guideline levels. Modulation has no significant role.	Further research. Methods to reduce exposures.	[115,116]
	MTHR. All topics. 2012	No increased cancer risk from wireless technologies. No robust evidence of harmful effects. No definite demonstrable effects in children.	No evidence for modulation effects on biological systems.	[118]
	IET. All topics. 2013–2014	No new robust evidence for adverse effects. Policy makers should consider all evidence including cost and benefits of mobile phone use.	Precaution “just in case”.	[120,121]
	ISLE of MAN. Phone Masts/ Children. 2009	No definite demonstrable effects on children.	Precautionary principle for mobile phone masts.	[123]
USA	ACS. Cancer. 2012	So far, no link between mobile phone use and cancer.	Further research especially in children and long term use.	[125]
	ACS. Cell Towers. 2013	No evidence that cell phone towers cause any health problems.		[126]
	FCC. All topics. 2013	No evidence for cancer or a variety of other problems, including headaches dizziness or memory loss	-	[128]
	FDA. All topics. 2012	Studies on biological changes were not replicated. No evidence for health problems in adults, children and teenagers.	Reduce exposures.	[130,131]
	NCI. Cancer. 2013	Studies have not shown a consistent link with cancers of the brain, nerves, or other tissues of the head and neck cancers.	Reduce exposures. More research, technology is changing rapidly.	[133]

#### 2.2.1. Australia

The Australian Radiation Protection and Nuclear Safety Agency (ARPANSA) [53] has issued more than 10 EME factsheets, in the area of non-ionizing electromagnetic fields in the frequency range 0–300 GHz and all of them can be downloaded from the website [54]. In the first factsheet (EME-1) on electromagnetic energy and its effects, ARPANSA stated [55]: *“The weight of national and international scientific opinion is that there is no substantiated evidence that exposure to low level RF EMF causes adverse health effects”*. Regarding the RF exposure standards (EME-4), ARPANSA stated [56]: *“…the SAR limit for mobile phone handset is 2 watts per kilogram of tissue (averaged over 10 g). The limit includes a significant safety factor….”*. Regarding base stations (EME-9), [57]: *“The weight of national and international scientific opinion is that there is no substantiated evidence that RF emissions associated with living near a mobile phone base station or telecommunications tower poses a health risk”*. Referring to mobile phones and children (EME-11) [58]: *“At present, there is insufficient evidence in the science to substantiate the hypothesis that children may be more vulnerable to RF EMF emissions from mobile phones than adults”….. “It is recommended…parents encourage their children to limit their exposure by reducing call time, by making calls where reception is good, by using hands-free devices or speaker options, or by texting”*. In the latest technical report #164 which was released in 2014, ARPANSA’s perspective [59]: *“the underlying basis of the ARPANSA RF exposure Standard (also referred to as RPS3) remains sound and that the exposure limits in the Standard continue to provide a high degree of protection against the known health effects of RF electromagnetic fields”* It is stated* “based on the in vitro/in vivo research, there is no evidence of a need for the reconsideration of the exposure limits in RPS3. However, the rationale for a precautionary approach may need to be clarified in the light of the growth in the body of knowledge over the last 10 years. The epidemiology of exposures to RF electromagnetic fields has not progressed with any dose-response relationships regarding carcinogenic and non-carcinogenic effect which would warrant significant changes to RPS3”*.

#### 2.2.2. Belgium 

The “Health, Food Chain Safety and Environment” (FPS) [60] is the scientific advisory body of the Belgian Superior Health Council (SHC) which prepares scientific advisory reports to guide political decision-makers and health professionals to guarantee and enhance public health. In 2011, FPS stated [61]: *“So far, it has not been proven that the radiation from mobile telephones is harmful to their users. But, on the foundation of current scientific knowledge, health risks relating to long-term, frequent mobile phone use cannot be ruled out”.* Nonetheless, more recently, in 2014 [62], the Superior Health Council advised *“…everyone to limit their exposure to mobile phone radiation”.* Referring to electromagnetic hypersensitivity: *“a nocebo effect plays a role (a negative effect is caused by negative expectations)”*. Further regulations came into effect [63]: *“As of 1 March 2014, mobile phones that are specially designed for young children may no longer be introduced to the Belgian market. The SAR value will have to be indicated along with other technical specifications, not only in the shop, but also for distance sales over the Internet.”*

#### 2.2.3. Canada

Health Canada [64] is responsible for helping Canadians maintain and improve their health and conducts its own research related to the biological effects of non-ionizing electromagnetic fields and develops regulations, guidelines and safety codes pertaining to radiation-emitting devices. After IARC classification of RF as class 2B carcinogen in 2011 [29], Health Canada stated in 2012 [65]: *“Health Canada reminds cell phone users that they can take practical measures to reduce their RF exposure”. “…also encourages parents to take these measures to reduce their children’s RF exposure from cell phones since children are typically more sensitive to a variety of environmental agents”. “With respect to cell phone towers, as long as exposures respect the limits set in Health Canada’s guidelines, there is no scientific reasons to consider cell phone towers dangerous to the public”. “Health Canada continues to monitor the science regarding RF exposure and would take action if future research establishes RF energy poses a health risk to Canadians”*]. Regarding Wi-Fi equipment, in 2013, Health Canada stated [66]: *“…there is no convincing scientific evidence that exposure to low-level radiofrequency (RF) energy from Wi-Fi causes adverse health effects in humans”*. Regarding the Safety Code 6 which limits exposures to RF, the Royal Society of Canada Expert Panel issued a statement in 2014 [67]: *“Canadians are protected from continuous exposure to multiple sources of RF energy when Safety Code 6 is respected”*.* “Since the last revision of Safety Code6 in 2009, no new adverse health effects have been established”*. 

#### 2.2.4. Finland 

The Radiation and Nuclear Safety Authority in Finland (STUK) [68] belongs to the Ministry of Social Affairs and Health in Finland. Its own research program (HERMO, 2004–2009) comprised of 13 different projects in which the investigators examined RF effects, especially on nervous system and sensory organs in addition to any possible detrimental effects on children and adolescents. A report issued in 2007 stated [69]: *“Using cell cultures, test animals, human subjects and mathematical models, the researchers said that their studies did not uncover any evidence of ill effects on health”*. Another study, which was part of HERMO, examined whether local exposure of human skin to RF-EMF will cause changes in protein expression in living people and concluded [70]: *“This is the first study showing that molecular level changes might take place in human volunteers in response to exposure to RF-EMF. Our study confirms that proteomics screening approach can identify protein targets of RF-EMF in human volunteers”*.

In a response to Finns and Finnish media on this issue, a statement was issued in 2009 [71]: *“The observed biological changes do not however indicate a health risk. At Turku University in Finland, the scientists… have not found any reproducible evidence that mobile phone radiation would have any cognitive influence”*. Regarding mobile phones, base stations and wireless networks, STUK issued a statement on 2013 [72]: *“The overall data published in the scientific literature to date do not show adverse health effects from exposure of radiofrequency electromagnetic fields below the guidelines or limits adopted in the Nordic countries. However, epidemiological studies on long-term exposure to radio waves from mobile phones are still limited, especially studies on children and adolescents. There are several ways to reduce exposure from mobile phones”*. In a more recent updated message in 2014, STUK reiterated [73]:*“Several studies, studies, in several countries, have tried to find out any other effects apart from heating. On the basis of the results obtained from the studies, it has not been possible to conclude that radiation from mobile phones would be detrimental to health”.* Regarding children,* “Parents are recommended to advice their children to use rather SMS messages than mobile phone calls ...restrict the number of calls and their duration ….use a hands-free that minimises the exposure of head ...” “STUK does not find it justifiable to totally prohibit children’s use of mobile phones. Mobile phones also create safety because they make children’s communication with parents easier”*.

#### 2.2.5. France

The French Agency for Food, Environmental and Occupational Health and Safety (ANSES, formerly AFSSET) [74] provides the authorities with the information on the risks and risk management strategies related to environmental issues, including electromagnetic fields. Based on the criteria used by IARC, a permanent independent expert group set up by the agency reviewed and evaluated all health effects of exposures to RF fields. In 2009, ANSES concluded [75]: *“If there is a risk associated with mobile telephony, it is low and related to mobile phones and not antennas. No scientific study has, indeed, could highlight the biological effects that would involve a health risk for populations living near mobile phone base stations, given their low level of emission of electromagnetic waves”.* The conclusions of the collective appraisal of ANSES were published more recently in 2013 [76]: (i) Among the biology studies, *“many well-conducted investigations showed no effects”.* (ii) Regarding neurological effects, the level of evidence is inadequate in humans on *“cognitive function, sleep, circadian rhythms, auditory function, neurodegenerative disorders such as Alzheimer’s disease in particular, amyotrophic sclerosis, multiple sclerosis and epilepsy (on the basis of a limited number of studies”.* (iii) Concerning other non-carcinogenic effects, the level of evidence is inadequate on *“male fertility, in utero development, teratogenesis, immune, endocrine, visual and cardiovascular systems”.* (iv) With respect to the potential carcinogenic effects, the evidence level is inadequate on *“development of gliomas in the general population, meningiomas, salivary gland and pituitary tumors, leukaemia, cutaneous and ocular melanomas, on cancer incidence and mortality (all types)”.* In an update, ANSES stated in 2013 [77]: *“This update has not brought to light any proven health effect and does not result any proposed new maximum exposure limits for the population”. “ANSES recommends limiting the population’s exposure to radiofrequencies—in particular from mobile phones—especially for children and intensive users, and controlling the overall exposure that results from relay antennas”*.

#### 2.2.6. Germany

##### 2.2.6.1. Commission on Radiological Protection (SSK)

The Commission on Radiological Protection (SSK) [78] was set-up by the Federal Ministry of the Environment in Germany to carry out a clear and transparent comparative assessment of the evidence of cancer risks and risk perception posed by electromagnetic fields (from static fields to ionizing radiation). The overall assessment regarding microwaves (RF) in 2011 [79]: *“…there is a discrepancy between the scientific evidence for cancer risk and the public’s risk perception”. “…including multinational studies, there is still no evidence for any link between mobile phone use and cancer. Some few epidemiological studies with inaccurate exposure data, memory bias and changes in mobile phone technologies during the study period reporting on possible brain cancer risk after more than 10 years of mobile phone use are not sufficiently reliable to justify changing this evidence classification”*.

##### 2.2.6.2. The German Mobile Telecommunication Research Program (DMF)

The German Mobile Telecommunication Research Program (DMF) [80,81] was launched in response to public concern about health effects of high frequency electromagnetic fields in the context of increasing mobile phone usage. The entire program (2002–2008) had 54 research projects in biology, epidemiology, dosimetry and risk communication. The Federal Office for Radiation protection (BfS) administered and managed the entire research program. The final reports of the research projects were evaluated by at least two independent experts. The conclusions from different projects in 2011 [82]: *“no evidence that electromagnetic fields can initiate or promote cancer”. “…no effects on the BBB even though they have used new methodological approaches”. “…no effect on visual, auditory, immune and cardiovascular system”. “…no effect on sleep and behavior in epidemiological studies and in field studies”. “…electrosensitivity most likely does not exist. “…highly improbable adverse effects on reproduction and development”. “…no higher sensitivity and a link between health effects in children/adolescents and exposure to mobile communications”.* Regarding risk perception and risk communication: *“…the frequency of anxiety and fears are linked to the extent and content of media reporting. Public concern about mobile phone base stations clearly exceeds that about mobile phones”*.

##### 2.2.6.3. Jülich 

Jülich Research Center/Institute [83] is a member of the Helmholtz Association of German Research Centers and is one of the largest interdisciplinary centers in Europe. Juelich published a report in 2009 on “Children’s health and RF EMF exposure” which was based on the scientific opinions of the internationally recognized experts and advisory experts from Australia, Austria, Belgium, Germany, Italy and Switzerland as well as from the discussions held in a series of workshops. Three main areas of children’s health were assessed: brain cancer, leukemia and cognition. In addition, dosimetry issues,* i.e.*, whether children absorb more power than adults when exposed to RF, were considered. The conclusions [84]: *“For children under 8 years, no conclusive evidence exists for the assumption that the SAR levels in children’s head is higher than for adults”. “There is no evidence showing that RF EMF exposure might induce brain cancer”. “…no substantive evidence for RF EMF exposure on cognitive performance and CNS functions of children”. “Overall, the review of the existing literature does not support the assumption that children’s health is affected by RF EMF exposure from mobile phones or base stations”*.

#### 2.2.7. Latin America

A multidisciplinary panel of researchers in 10 Latin American countries (Argentina, Bolivia, Brazil, Chile, Colombia, Ecuador, Panama, Paraguay, Peru, and Venezuela) critically reviewed the peer-reviewed scientific papers on the possible biological and health impact of mobile communications devices to address the increasing concerns of the general public in those countries. The project had a special emphasis on the studies conducted in Latin American countries. The general conclusions published in 2010 were [85]: *(i) inadequate evidence or a lack of consistent and validated evidence from experimental in vitro studies, at least, when RF exposures were below recommended safety levels, (ii) no convincing evidence from acute or chronic effects on physiological and biochemical parameters in animal studies, (iii) no significant effects of cell phone usage or reasonable proximity to radiating antennas of base stations, (iv) human provocation studies found no significant effects of cell phone usage or living near base stations,* and *(v) human epidemiological studies have indicated non-significant association with neurodegenerative, cardiovascular and reproductive disorders, behavioral changes, nonspecific symptoms, cataracts, morbidity, mortality, well-being and health status”.* The panel also stressed that attention should also be paid to the beneficial uses of wireless communication devices such as *“contacting the police in case of robbery, theft, family violence, malfunction of vehicles on the road”*.

#### 2.2.8. Netherlands (Health Council)

The Health Council of the Netherlands [86] is an independent scientific advisory body and a member of the European Science Advisory Network for Health and the International Network of Agencies for Health Technology Assessment. The standard committee members of the Council were selected form a multidisciplinary group of independent experts to provide advice to the Ministers and Parliament in the field of public health and healthcare research. 

The annual update from the Council in 2009 indicated [87]: *“The picture that emerges from the available scientific evidence is that there is no causal link between exposure to radiofrequency electromagnetic fields and the occurrence of medically unexplained physical symptoms. However, there is a link between the symptoms and “assumed” exposure and with that very probably a link to risk perception. Nevertheless, the symptoms do exist and require a solution”*. Regarding the influence of RF on children’s brains, the Council concluded in 2011 [88]: *“…still relatively limited available data do not indicate any effects on the development of the brain or on health”* and *“…no reason to recommend different exposure limits for children than for adults”*. More recently, in 2013, the Council followed a priori defined protocol, strength and weaknesses in the studies, consistency in the results, plausibility and a standardized scoring system. Part 1 of the report dealt with the epidemiology of tumors in the head and the conclusions were [89]: *“…despite substantial research efforts, there is still insufficient clarity and consistency regarding a possible association between mobile phone use and an increased risk of tumours in the brain and other regions of the head. There is some weak and inconsistent evidence for an association between prolonged and intensive use of a mobile phone and an increased incidence of gliomas. This is most likely explained by various types of bias and chance, but it cannot be excluded that there is a causal relation. For the other types of tumours, including meningiomas and acoustic neuromas, indications for an increased risk are much weaker or completely absent”*.

#### 2.2.9. New Zealand

##### 2.2.9.1. National Radiation Laboratory (NRL) 

The National Radiation Laboratory (now, National Centre for Radiation Science) [90] provides advice on ionizing and non-ionizing radiation to benefit New Zealand’s environment, industries and the general public. In the information sheet issued on the safety of cell phones, in 2008 [91], NRL stated: *“The balance of current research evidence suggests that exposures to the radiofrequency fields produced by cellphones do not cause health problems provided they comply with international guidelines”.* Regarding the use of cellphones by children,* “a matter for informed choice by parents, bearing in mind that cellphones can improve personal safety”.* NRL suggested several methods to reduce individual’s exposure to RF from mobile phones.

##### 2.2.9.2. Cancer Society of New Zealand 

The Cancer Society of New Zealand [92] provides a range of information including booklets, leaflets, tapes, videos and books on different types of cancer, diagnosis, treatment, and advice for everyone in New Zealand. The society issued an information sheet on mobile phones and cancer in 2010 [93]: *“There is no clear evidence, at this time, that short-term mobile use can cause cancer. This is an area that is changing fast, and the research is ongoing”.* Therefore, the Cancer Society advices people to limit exposure when practically possible and these include *“young children should not use mobile phones unless they really have to”*.

#### 2.2.10. Nordic Countries

The health and nuclear safety authorities in five Northern European countries (Denmark, Finland, Iceland, Norway, Sweden) have joined to form the Nordic Radiation Safety Authorities and issued a common statement on radiofrequency electromagnetic fields exposure to the general public in 2009 [94]: *“there is no scientific evidence for adverse health effects caused by radiofrequency field strengths in the normal living environment at present”. “…in terms of overall public exposure, mobile phones are a much more significant source of radiofrequency radiation than fixed antennas. If the number of fixed antennas is reduced, mobile phones will need to use higher power to maintain their connection, thereby the exposure of the general public may increase”*. More recently, in 2013, another statement was issued regarding exposures from mobile phones, base stations and wireless networks [95]: *“The overall data published in the scientific literature to date do not show adverse health effects from exposure of radiofrequency electromagnetic fields below the guidelines or limits adopted in the Nordic countries”,“…no need to further limit exposure from these radiowave sources”*.

#### 2.2.11. Norway 

The Ministry of Health and Care Services and, the Ministry of Transport and Communications had commissioned the Norwegian Institute of Public Health [96] to appoint an expert committee. The committee’s conclusions in 2012 were [97]: *“A large number of studies have examined the possible effects of exposure to weak RF fields (i.e., exposure within the ICNIRP’s reference values). The studies have been performed on cells and tissues, and in animals and humans. The effects that have been studied apply to changes in organ systems, functions and other effects. There are also a large number of population studies with an emphasis on studies of cancer risk. The large total number of studies provides no evidence that exposure to weak RF fields causes adverse health effects. Some measurable biological/physiological effects cannot be ruled out”. “Overall, the uncertainty in risk assessment is therefore small”.* The committee also outlined three levels of precaution that can be exercised when handling a risk, depending on the nature of the risk, the severity, uncertainty in the assessment, and any consequences.

#### 2.2.12. Spain 

The Scientific Advisory Committee on Radio Frequencies and Health (Comité Cientifico Asesor en Radio-frecuencias y Salud, CCARS) [98] is an independent institution composed of acknowledged experts in medicine, physics, chemistry, biology, law and other related disciplines. Its mission is to provide judgment, information and advice to Public Administration regarding questions concerning RF and health. In 2009, CCARs concluded [99]: *“A review of the literature that the use and exposure of adults to mobile phones over a period of less than 10 years is not associated with an increased risk of brain tumor, and that the results of recent scientific research do not justify changes in Spain’s exposure limits”* which are currently based on ICNIRP guidelines. Recently, in 2011, the CCARS has drafted a report analyzing the possible health effects of Wi-Fi systems in school children [100] which *“…overwhelmingly concludes that, at least to date, there is absolutely no scientific evidence that exposure to the low emission levels of these systems produces adverse health effects in school children. There are therefore no reasons to suppose that such Wi-Fi systems should not be used by schoolchildren and other population groups”*.

#### 2.2.13. Sweden

##### 2.2.13.1. Swedish Radiation Safety Authority (SSM/SSI)

The Swedish Radiation Protection Agency (SSM/SSI) [101] belongs to the Ministry of the Environment in Swedish Government and works proactively and preventively in order to protect people and environment from the undesirable effects of radiation. An international independent expert group was appointed by the Agency to evaluate scientific developments and provide advice on the possible health effects of electromagnetic fields. 

The revised edition of the 5th report was published in 2008 and the conclusions were [102]: *“In general, the recent, methodologically more rigorous studies do not replicate the positive findings from smaller, less rigorous studies published a few years ago, but a few positive effects are reported. Two national Interphone publications are based on very small numbers and do not change the overall assessment, and two published meta-analyses provide little additional information”*. 

The 6th report was published in 2009 and the conclusions were [103]: *“There are no new positive findings from cellular studies”*. *“...animal studies have not identified any clear effects on any of a number of different biological endpoints”*. *“Many epidemiological studies of mobile phone use and brain tumour risk observe effect estimates below unity …especially for short term mobile phone use, which if true would imply a protective effect”*. This is the first time any expert group used the term “protective effect” for the observed risk estimates which were below unity between mobile phone use and cancer. Vijayalaxmi and Prihoda [27] and Vijayalaxmi *et al*. [28] have proposed a hypothesis of RF-induced “adaptive response” to describe such protective effect. Kundi [104] suggested “systematic bias” for these reduced risk estimates.

The 7th report was published in 2010 and the conclusions were [105]: *“…for up to about ten years of mobile phone use associations with brain tumour risk are unlikely*. *For longer duration of use, for specific subtypes of cancer, and for children and adolescents data are sparse or non-existing, and conclusions are less certain. Available data do not indicate any risks related to exposure to RF from base stations or radio or TV antennas”*. 

The 8th report was published in 2013 (after IARC classification of RF as class 2B carcinogen) [106]. It was an update on key issues covered in both 2011 and 2012 and the following were the conclusions. (i) Cell studies: *“Recent data from laboratory studies related to cancer do not seem to support the conclusion of IARC that RF EMF is a possible carcinogen”.* (ii) Animal studies: *“No increased cancer risks were observed”.* (iii) Human epidemiological studies: *“In new epidemiological studies, some protective as well as some adverse effects have been observed in child development, reproductive health, multiple sclerosis, cognitive decline in elderly, auditory functions, bone mineralisation and hypertension, but methodological limitations prevent from firm conclusions in terms of causal associations”.* (iv) Self-reported electromagnetic hypersensitivity and symptoms: *“the new epidemiological studies … indicate the absence of a risk … on health-related quality of life”*. 

The latest 9th report was published in 2014 [107] and the following were the conclusions. (i) Cell studies: *“most of the studies reported do not support an effect of RF EMF on DNA damage or cell death, and only minimal effects on protein expression”*. (ii) Animal studies: *“the majority of the recent animal studies still lack of clear working hypothesis*. *Weak indications of possible effects of oxidative stress and brain function including behavior”*. (iii) Human studies: *“… no demonstrable effect on cognitive function…”*. (iv) Epidemiological studies: *“The glioma results ...arein contradiction with the recent and previous time-trend studies …”*. *“No increase in salivary gland tumors and skin cancer in the head and neck region in relation to mobile phone use*. *Many non-cancer outcomes and self-reported hypersensitivity studies have considerable/severe limitations and thus no firm conclusions can be drawn”*.

##### 2.2.13.2. Swedish Research Council for Working Life (FAS)

The Swedish Research Council for Working Life and Social Research (FAS) [108] was commissioned by the Swedish Ministry of Health and Social Affairs to monitor, document and report on the state of research related to human health effects of exposure to electromagnetic fields. The conclusions in 2012 [109]: *“Overall, the data on brain tumor and mobile telephony do not support an effect of mobile phone use on tumour risk, in particular when taken together with national cancer trend statistics throughout the world”. “Extensive research for more than a decade has not detected anything new regarding interaction mechanisms between radiofrequency fields and the human body and has found no evidence for health risks below current exposure guidelines. While absolute certainty can never be achieved, nothing has appeared to suggest that the since long established interaction mechanism of heating would not suffice as basis for health protection”*.

#### 2.2.14. Switzerland 

The federal office of the environment (FOEN) [110] in Switzerland is responsible for environmental monitoring and, appropriate use and protection of natural resources. The status of scientific knowledge regarding potential risks to health due to exposure to high-frequency non-ionizing radiation (100–300 GHz) has been published earlier. However, the focus of the latest up-dated report in 2012 was on exposure to high-frequency radiation from fixed installations such as broadcasting transmitters and mobile telephone base stations in experimental field studies, epidemiological studies of population groups in their everyday environment, and short-term exposure studies under controlled conditions in the laboratory. The conclusions [111]: *“…no new confirmed health effects of exposure to high-frequency fields from transmitters were observed in the dose range below the recommended reference levels of the International Commission for Non-Ionizing Radiation Protection (ICNIRP), and thus below the ambient limit values specified in the ONIR. From the scientific point of view, this means that protection against acute effects is assured as before”. “In view of the fact that there are gaps in the available data, the absence of proof of health risks does not automatically also mean proof of their absence. From the scientific point of view, a cautious approach in dealing with non-ionising radiation is still called for. There remains a need for extensive research into the potential long-term effects”*.

#### 2.2.15. Tanzania 

The Tanzania Communications Regulatory Authority (TCRA) [112] is a statutory regulatory body responsible for regulating the communications and broadcasting sectors in Tanzania. It was established under the TCRA Act No. 12 of 2003 which merged the Tanzania Communications Commission (TCC) and the Tanzania Broadcasting Commission (TBC. Its mission is to develop an effective and efficient communications regulatory framework, promote efficiency among the communications services providers, and protect consumer interests with an objective of contributing to socio-economic and technological development in the United Republic of Tanzania. A public notice issued in 2010 [113] stated: *“…no substantial evidence that the use of communications equipment causes harmful health effects. Consumers should continue to have confidence in the many benefits of modern technology including mobile telephony, TV and computers which are used by people globally. This information has been prepared on the basis of substantiated research”*.

#### 2.2.16. United Kingdom

##### 2.2.16.1. Health Protection Agency (Public Health England, PHE) 

The Health Protection Agency (formerly National Radiological Protection Board) [114] had set up an independent advisory group on non-ionizing radiation (AGNIR) to evaluate the health effects from RF emitted from various sources in the environment (100–300 GHz) and to review work on the biological effects of non-ionizing radiation relevant to human health and to advice on research priorities. The general conclusion in April 2012 [115]: *“…although a substantial amount of research has been conducted in this area, there is no convincing evidence that RF field exposure below the guideline levels causes health effects in adults or children”.* The group recommended further research in all areas of RF exposure.

Several updates were published in 2013 [116]. Regarding the measurements made at base stations in UK*: “…exposure at publicly-accessible locations near to base stations is very much below the ICNIRP guidelines”.* Referring to mobile telephony and health: *“It is not possible to show that reducing an exposure within the ICNIRP guidelines gives any specific health benefit. Nevertheless, IEGMP felt that people buying mobile phones should have the information to enable them to choose to reduce their exposure if they so wished”.* With respect to Wi-Fi equipment: *“On the basis of current scientific information, exposures from Wi-Fi equipment satisfy international guidelines. There is no consistent evidence of health effects from RF exposures below guideline levels and no reason why schools and others should not use Wi-Fi equipment”*.

##### 2.2.16.2. The Mobile Telecommunications and Health Research (MTHR)

The Mobile Telecommunications and Health Research (MTHR) [117] program was established in 2001 as part of UK government’s response to the recommendation of the independent expert group on mobile phones, which was chaired by Sir. William Stewart. The program had several research projects and the final report was published in 2012 [118]. The conclusions were: *“Taken together, the studies … do not suggest that exposure to mobile phone signals is associated with an increased risk of cancer”. “… a substantial body of evidence that modulation does not play a significant role in the interaction of radiofrequency fields with biological systems”*.

##### 2.2.16.3. Institute of Engineering and Technology (IET) 

The Institution of Engineering and Technology (IET) [119] had established an expert biological effects policy advisory group (BEPAG) to review the possible health effects of electromagnetic fields and issues reports/fact-files/position statements periodically. The conclusions in the latest report in 2013 were [120]: *“With respect to base stations, the studies have limitations but do not find evidence that risks of childhood cancers are greater in the vicinity of mobile-phone masts”. ”…there is no sign that the incidence of cancer is increasing in response to wireless technologies, as would be expected if there were a link”. “There have been claims that heavy users of mobile phones experience more headaches, loss of memory and insomnia. At present it has not been shown that these symptoms are caused by EMFs. Given the uncertainties, it is understandable that people may wish to reduce their personal exposures “just in case”*. The update published in 2014, IET stated [121]: *“In summary, the absence of robust new evidence of harmful effects of EMFs in the past two years is again reassuring and is consistent with our findings over the past two decades. The widespread use of electricity and telecommunications has demonstrable value to society, including numerous health benefits. BEPAG is of the opinion that it remains important that these factors, along with the overall scientific evidence, should be taken into account by policy makers when considering the costs and benefits of both the implementation of any precautionary approaches to public exposure and also in the development of public-exposure guidelines”*.

##### 2.2.16.4. Isle of Man

The Chief Minister of Isle of Man [122] in UK had set up a committee to review the scientific publications on health impact of mobile telephone masts. The recommendations of the committee in 2009 [123] were: *“…although there are no definite demonstrable effects on children, it would be prudent not to site base stations in locations where children are likely to be exposed to the beams for a long duration”.* The committee also recommended *“The use of precautionary principle in the siting of mobile phone masts”*.

#### 2.2.17. United States of America

##### 2.2.17.1. American Cancer Society (ACS) 

The American Cancer Society (ACS) [124] is a nationwide voluntary health organization in USA which provides information to the general public from its National Cancer Information Center. Regarding cell phones, the position of ACS in 2012 [125]: *“…most studies published so far have not found a link between cell phone use and the development of tumors. However, these studies have had some important limitations that make them unlikely to end the controversy about whether cell phone use affects cancer risk”. “…it is important that the possible risk of cell phone exposure continue to be researched using strong study methods, especially with regard to use by children and longer term use”*. Regarding the cell phone towers in 2013 [126]*: “So far, there is no evidence in published scientific reports that cell phone towers cause any other health problems”.* Overall, ACS recommends limit on cell phone use by adults and children.

##### 2.2.17.2. Federal Communication Commission (FCC)

All wireless devices sold in the USA should go through a formal FCC [127] approval process. In answers to frequently asked questions, in 2013, FCC stated [128]: *“FCC requires wireless phones to have SAR levels no greater than 1.6 watts per kilogram”*. *“There is no scientific evidence that proves that wireless phone usage can lead to cancer or a variety of other problems, including headaches, dizziness or memory loss”*.

##### 2.2.17.3. Food and Drug Administration (FDA)

The Food and Drug Administration (FDA) [129] belongs to the Federal RF Interagency Work Group (other members: National Institute for Occupational Safety and Health; Environmental Protection Agency; Federal Communications Commission; Occupational Safety and Health Administration; National Telecommunications and Information Administration) and has the responsibility for different aspects of RF safety at the federal level. In answers to questions related to cell phone use and children, in 2012, FDA stated [130,131]: *“While some researchers have reported biological changes associated with RF energy, these studies have failed to be replicated. The majority of studies published have failed to show an association between exposure to radiofrequency from a cell phone and health problems”. “The scientific evidence does not show a danger to any users of cell phones from RF exposure, including children and teenagers. The steps adults can take to reduce RF exposure apply to children and teenagers as well. Reduce the amount of time spent on the cell phone”*.

##### 2.2.17.4. National Cancer Institute (NCI)

The National Cancer Institute [132] had issued a fact sheet in 2013 which stated [133]: *“…studies thus far have not shown a consistent link between cell phone use and cancers of the brain, nerves, or other tissues of the head or neck. More research is however needed because cell phone technology and how people use cell phones have been changing rapidly”*.

## 3. Comments

The opinions of a total of ~35 expert groups and health authorities were published during the 2008–2014 and, the vast majority expressed the opinion that there was inadequate evidence for increased biological and health risks in humans exposed to RF fields emitted from wireless communication devices (and base stations in some reports). Because of the absence of sufficientlong-term RF exposure studies and in view of the long latency period for certain parameters, such as development of cancers and neurological diseases, almost all of the recent reports recommended pre-cautionary measures to reduce exposure levels (decreasing the number of calls, call time and using hands-free-devices). Parents were particularly advised that their children should use mobile phone only when absolutely necessary. This precaution was introduced, especially, after IARC evaluation of RF as a possible carcinogen in class 2-B. Some reports have also mentioned that mobile phones play an important role in cases of accidents, malfunction of vehicles on the road, emergency, robbery, theft,* etc.*

Some “negative” comments. (i) The selection procedure used to select the members in expert groups (EGs) in various countries was neither clear nor transparent. (ii) It was difficult and almost impossible to verify “no conflict of interest” of the members in the EGs. (iii) The criteria used for evaluations were not sufficiently described in some reports. (iv) Some members participated in more than one expert group (for example, the experts in SSI report were also some members of ICNIRP). (v) Several EGs did not consider the health risks associated with mobile phone base stations. (vi) There was an apparent “bias” in selecting the papers for evaluation: the reports that support their analysis were reviewed and left out those that contradict their conclusions.

Some “positive” comments. (i) Members chosen in EGs had expertise in all aspects of RF research, such as dosimetry, biology, epidemiology, statistics,* etc.* (ii) All peer-reviewed scientific publications were considered in the evaluation process. (iii) Attention was been paid to the detailed description given in the publications, *viz*., dosimetry, exposure set-up/parameters, methods/protocols used in the study, sample size, confounding factors,* etc.* (iv) The evaluations were based on the same peer-reviewed scientific publications. Generally, the Belgian Superior Health Council was often criticized for emphasizing too much on the precautionary principle and providing information that was not scientifically sound while the ICNIRP and Health Council in Netherland were often accused of insufficiently applying the precautionary principle. Overall, IARC had paid special attention on “conflict of interests” of the members in the EGs and, the evaluations were based on extensive and exhausting review of scientific literature performed by a great number of experts according to a well described and rigid procedure as well as “face to face” personnel discussions and deliberations. Further, the conclusions were also voted on by members of all expert groups [29]. Nonetheless, Wiederman *et al.* [134] commented *“There should be some concern that there are working group members who are the very researchers assessing the quality of their own studies”* and suggested *“select working group members who are not involved in the EMF field to conduct a truly independent review”*. The comment implied “conflict of interest” among IARC expert working group members and hence, compromised the credibility of IARC conclusion. However, the suggestion may lead to other credibility problems since the “experts” should have “expertise/peer-reviewed publications” in or closely related field that is evaluated. 

## 4. Conclusions

During the last several decades, researchers have been evaluating the impact of* in vitro* and* in vivo* RF exposure in animal and human cells. The overall data used for scientific evaluation as well as the knowledge gained are more extensive now than ever before. Nonetheless, it is important to distinguish between an “adverse effect” and a “biological effect”. The IEEE [13] defined an adverse effect as *“A biological effect characterized by a harmful change in health that is supported by consistent findings that the effect was published in the peer-reviewed scientific literature, the evidence of the effect being demonstrated by independent laboratories and, where there is consensus in the scientific community that the effect occurs for the specified exposure conditions”* and, the biological effect as *“alterations of the structure, metabolism, or functions of a whole organism, its organs, tissues, and cells. Biological effects can occur without harming health and can be beneficial. Biological effects also can include adaptive responses”.*

After the IARC classification of RF as class 2B carcinogen, WHO also stated eloquently and precisely in 2011 [135]: *“To date, research does not suggest any consistent evidence of adverse health effects from exposure to radiofrequency fields at levels below those that cause tissue heating. IARC classified electromagnetic fields as possibly carcinogenic to humans (Group 2B), a category used when a causal association is considered credible, but when chance, bias or confounding cannot be ruled out with reasonable confidence”*.

It is significant that the guidelines recommended by the international organizations [12,13,14] included a large safety margin to limit exposures to electromagnetic fields and, these were based on the “established” “adverse” effects, *viz.*, electro-stimulation in the case of low frequencies and, whole-body and tissue heating in the case of high frequency RF fields. Hence, it is important for the international and national expert groups to recognize the difference between “adverse” and “biological” effects of RF exposure while relaying the scientific evidence to the authorities in order to formulate the necessary exposure guidelines and to provide accurate information to the general public.
